# Chromosomal Microarray Study in Prader-Willi Syndrome

**DOI:** 10.3390/ijms24021220

**Published:** 2023-01-07

**Authors:** Merlin G. Butler, Waheeda A. Hossain, Neil Cowen, Anish Bhatnagar

**Affiliations:** 1 Department of Psychiatry and Behavioral Sciences, University of Kansas Medical Center, 3901 Rainbow Blvd., MS 4015, Kansas City, KS 66160, USA; 2 Soleno Therapeutics, Inc., Redwood City, CA 94065, USA

**Keywords:** Prader-Willi syndrome (PWS), high-resolution chromosomal microarray, PWS molecular genetic classes, typical 15q11-q13 deletion subtypes, maternal disomy 15 subclasses, atypical PWS genetic findings, DESTINY PWS

## Abstract

A high-resolution chromosome microarray analysis was performed on 154 consecutive individuals enrolled in the DESTINY PWS clinical trial for Prader-Willi syndrome (PWS). Of these 154 PWS individuals, 87 (56.5%) showed the typical 15q11-q13 deletion subtypes, 62 (40.3%) showed non-deletion maternal disomy 15 and five individuals (3.2%) had separate unexpected microarray findings. For example, one PWS male had Klinefelter syndrome with segmental isodisomy identified in both chromosomes 15 and X. Thirty-five (40.2%) of 87 individuals showed typical larger 15q11-q13 Type I deletion and 52 individuals (59.8%) showed typical smaller Type II deletion. Twenty-four (38.7%) of 62 PWS individuals showed microarray patterns indicating either maternal heterodisomy 15 subclass or a rare non-deletion (epimutation) imprinting center defect. Segmental isodisomy 15 was seen in 34 PWS subjects (54.8%) with 15q26.3, 15q14 and 15q26.1 bands most commonly involved and total isodisomy 15 seen in four individuals (6.5%). In summary, we report on PWS participants consecutively enrolled internationally in a single clinical trial with high-resolution chromosome microarray analysis to determine and describe an unbiased estimate of the frequencies and types of genetic defects and address potential at-risk genetic disorders in those with maternal disomy 15 subclasses in the largest PWS cohort studied to date.

## 1. Introduction

Improved genetic testing has been developed over the past four decades and proven helpful to genetically confirm the diagnosis of Prader-Willi syndrome (PWS). The first advances in the early 1980s included high-resolution chromosome karyotypes that led to identification of the first microdeletion seen in PWS involving the 15q11-q13 region and reported by Ledbetter et al. [1]. In the late 1980s the discovery of DNA markers of genes identified in the 15q11-q13 region led to development of commercially available fluorescent in situ hybridization (FISH) in the early 1990s based on fluorescently labeled DNA probes hybridized usually by a single test probe to identify the deletion if the DNA sequence or structure is missing and a single normal control probe in the chromosome outside of the deletion region and may be with a different color visualized microscopically in the non-deleted region [2]. This method resulted in further discoveries of small deletions at the chromosome level for dozens of microdeletion syndromes besides PWS.

Comparative genomic hybridization (CGH) was developed and expanded as an array-CGH method to be used clinically in the 2000s. It provided an interface between thousands of DNA probes and cytogenetics by isolating DNA from the patient under study and normative controls, then fluorescently labeling the DNA aliquots differently (e.g., green or red). When equal labelled DNA quantities from a patient and a normal control with a different fluorescent color were mixed, the resulting computer-generated signals involving multiple probes covering all of the human chromosomes could detect deletions or duplications on each chromosome (e.g., red represented a deletion; green represented a duplication and yellow represented normal) [3].

Further research with the use of copy number and single nucleotide polymorphism DNA probes led to development of high-resolution chromosomal microarrays over the past 10 years were helpful in identifying small deletions or duplications and uniparental disomy as seen in Prader-Willi syndrome and/or other syndromes such as Angelman, Williams and Smith-Magenis along with dozens of other congenital malformation disorders in which the cause was previously unknown [4]. Prior to advances in genetic testing, the detection of subtle genetic anomalies with high precision would not be possible, particularly in identifying clinical genetic syndromes and confirmation of patients presenting with features of a microdeletion syndrome. In addition, regions of homozygosity (ROH) or absence of heterozygosity (AOH) of 3 Mb in size were helpful in determining identical by decent or consanguinity and later loss of heterozygosity (LOH) of 8 Mb in size was proven useful for identification of uniparental disomy such as maternal disomy 15 in PWS [5,6].

Prader-Willi syndrome is recognized as the first example in humans of errors in genomic imprinting and generally due to lack of expression of a cluster of paternally inherited genes on chromosome 15q11-q13 generally from a paternal deletion or by uniparental maternal disomy 15 [6] (e.g., Butler 2016). PWS is recognized by severe hypotonia in infancy with a poor suck, feeding difficulties and failure to thrive, hypogonadism/hypogenitalism, cryptorchidism, short stature and small hands and feet due to growth and other hormone deficiencies and developmental delay. During early childhood, mild intellectual disability is noted along with food seeking and hyperphagia leading to obesity, if not externally controlled. Obesity and behavioral problems present in childhood includes anxiety, temper tantrums, skin picking and compulsions and can contribute to other comorbidities and clinical findings [7]. PWS accounts for one in 15,000 to 20,000 live births with over 400,000 individuals worldwide [8]. 

**Figure 1:**
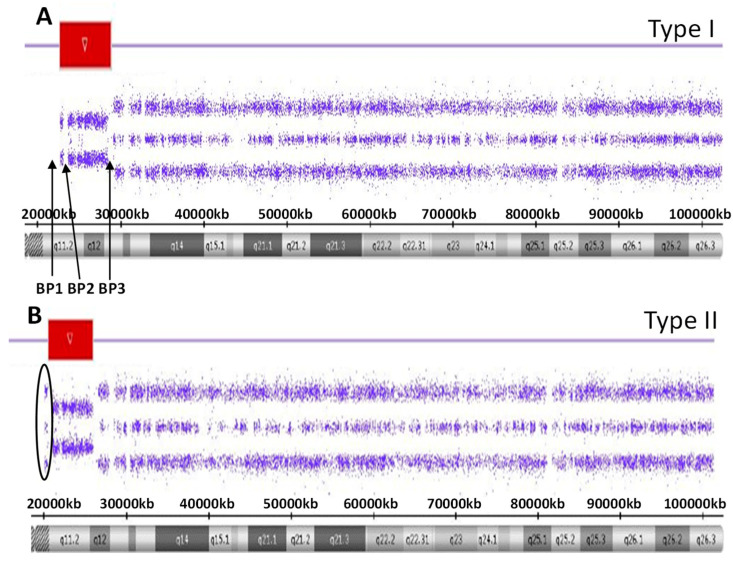
High-resolution chromosome microarray results for the larger typical 15 q11-q13 Type I deletion involving breakpoints BP1 and BP2 (**A**) while the smaller typical 15q11-q13 Type II deletion involving breakpoints BP2 and and BP3 (**B**). The red-colored bars represent the deletion region for both the Type I and Type II deletions.

**Figure 2:**
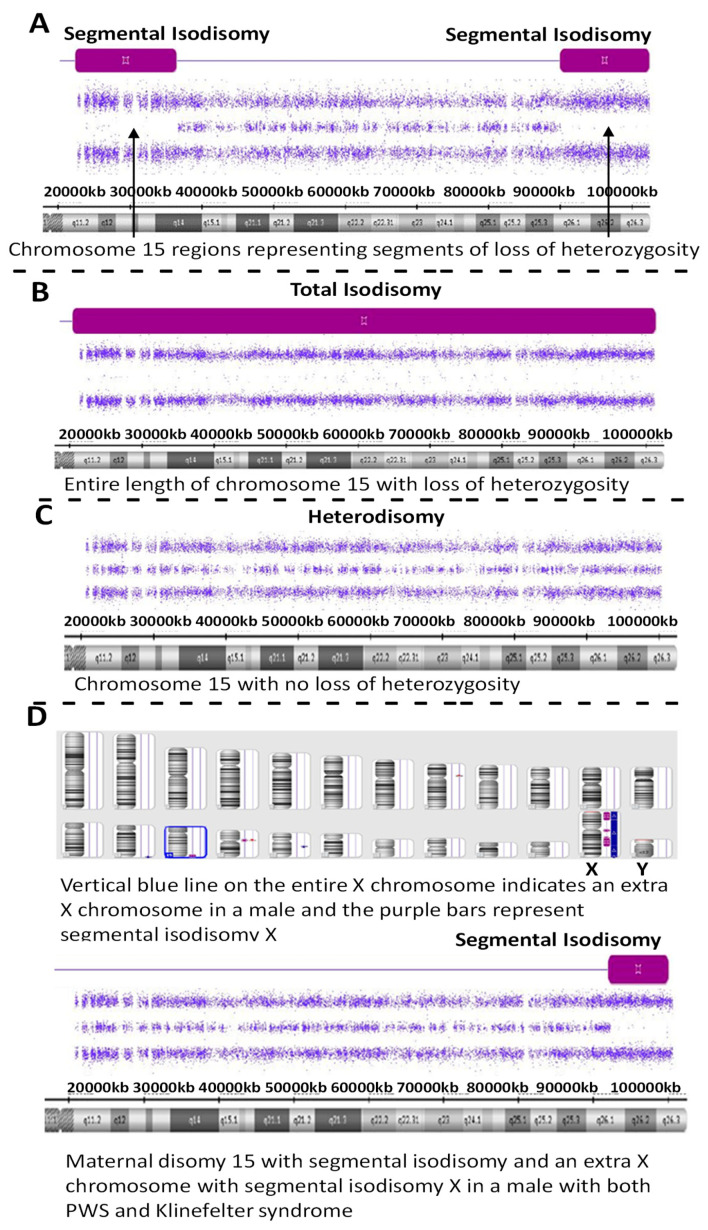
High-resolution chromosome microarray results showing examples of maternal disomy 15 in four PWS participants (**A**–**D**). Segmental isodisomy 15 is represented by segments of loss of heterozygosity as a purple-colored bar and found in (**A**,**D**) while total isodisomy 15 is seen in (**B**). Maternal heterodisomy 15 is shown in (**C**) with no loss of heterozygosity. (**D**) represents a PWS participant with segmental isodisomy 15 and segmental isodisomy X but with an extra X chromosome indicating the presence of Klinefelter syndrome, as well.

There are five recognized chromosome 15q breakpoints (BP1, BP2, BP3, BP4 and BP5) with two typical paternal 15q11-q13 deletions causing PWS and classified as a larger Type I or smaller Type II deletion including the Prader-Willi syndrome critical region (PWSCR). The Type I deletion involves a proximal 15q11 breakpoint BP1 and a distally located 15q13 breakpoint BP3 while the smaller Type II deletion involves a second proximal 15q11 breakpoint BP2 but with the same distal breakpoint BP3 [9,10] (see Figure 1). An average size of the larger Type I deletion is approximately 6 Mb, while the smaller Type II deletion is approximately 5.5 Mb. The larger deletion encompasses four non-imprinted genes (TUBGCP5, CYFIP1, NIPA1 and NIPA2) that reside between BP1 and BP2. Those individuals with PWS and the larger Type I deletion often have more learning and behavioral problems specifically compulsions, maladaptive behaviors and self-injury along with lower cognitive skills when compared to those with PWS having the smaller Type II deletion [11,12]. Specific clinical differences have also been reported in those with the second major cause of PWS, that is, maternal disomy 15 (see Figure 2). Those with PWS and maternal disomy 15 have a higher verbal intelligence quotient (IQ) than those with the paternal 15q11-q13 deletions and less self-injury but with more psychosis and autism [6,13,14].

The PWS cohort was recruited by DESTINY PWS (ClinicalTrials.gov number NCT03440814), an international, randomized, double-blind, placebo-controlled, parallel-group, Phase 3 study comparing diazoxide choline extended-release tablet (DCCR) to placebo in individuals with PWS [15]. The study enrolled males and females with PWS, aged 4 years and older with hyperphagia, weighing between 20 and 135 kg, in a stable care setting, at 29 sites in the US and UK. High-resolution chromosomal microarrays to analyze DNA samples from individuals enrolled in the study were used to confirm their genetic class. 

This report and review will summarize the results of the DESTINY PWS clinical trial and characterize the genetic findings in a large PWS cohort. The importance to increase awareness and confirmation of genetic defects causing PWS will be stressed and discussed including genetic mechanisms and relationships with reported chromosome 15 recessive genes depending on specific PWS molecular subtypes and subclasses with potential novel genetic changes as a component of the PWS diagnosis. This study may stimulate additional research to further understand the genetic causation of PWS, clinical treatment and surveillance along with genetic counseling purposes.

## 2. Results

Of the 154 individuals with PWS, 87 (56.5%) showed a 15q11-q13 deletion with 35 (22.7%) having the larger typical 15q11-q13 Type I deletion subtype. Fifty-two individuals (33.8%) had the smaller typical 15q-q13 Type II deletion subtype (see Figure 1). One (ID #20-055176) of these PWS subjects had the typical Type I deletion, but also a small duplication at 15q13.3 (433 kb in size) that contained two genes (OTUD7A and CHRNA7) residing between breakpoints BP4 and BP5. Deletions of this region are associated with neurodevelopmental problems and seizures (www.omim.org). Of those with the Type II deletion, one (ID #20-058046) had a small duplication at 15q13.1-q13.2 (187 kb in size) and contained two poorly characterized genes (TJP1 and GOLGA8) residing between breakpoints BP4 and BP5. 

**Figure 3:**
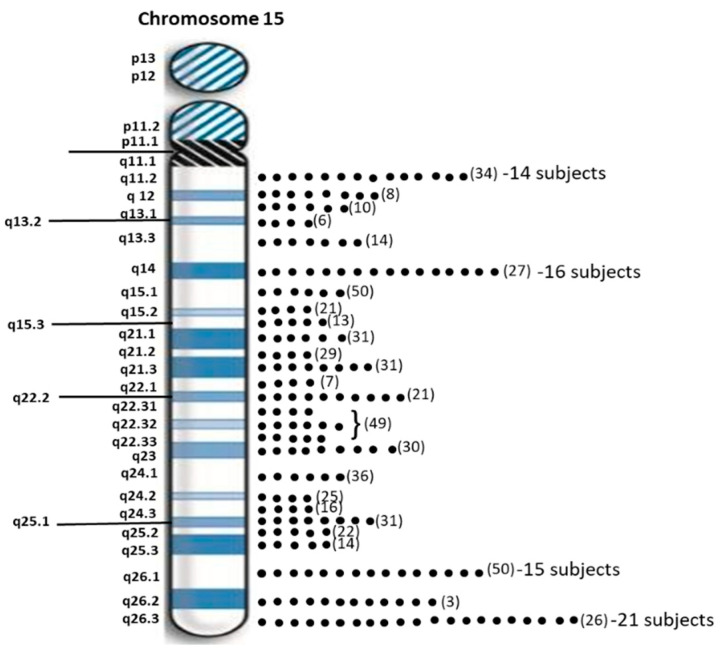
Distribution of chromosome 15 bands involved in segmental isodisomy 15 found by high-resolution microarray analysis and dense genotyping of chromosome 15 in our study of 154 PWS participants with maternal isodisomy 15 seen in 38 subjects. The number of protein coding genes in parentheses is noted per chromosome 15 band. The individual dots represent individual participants with that band involved in segmental or total maternal isodisomy 15. Chromosome 15q26.3 band was most often found in 21 PWS participants followed by 15q14 band in 16 participants.

Sixty-two individuals (40.3%) with PWS had maternal disomy 15 with segmental isodisomy 15 in 34 subjects (22 females, 12 males) or 54.8 percent due to normal cross-over events in female meiosis I with gene segregation (see Figure 2). The average size of the total LOH isodisomic region was 27.06 Mb and each LOH varied in size from 5.57 to 52.22 Mb. The average size of individual isodisomic regions was 18.40 Mb. Twenty-two of these subjects showed one LOH segment, ten showed two separate LOH segments and two showed three LOH segments. LOHs were seen throughout chromosome 15 involving the proximal, middle or distal long arm. Chromosome 15 bands at the terminal (15q26.3; 21 PWS subjects), middle (15q14; 16 PWS subjects) and proximal (15q26.1; 15 PWS subjects) regions were most often involved (see Figure 3). Two subjects had only a proximal LOH segment, four subjects had only a middle LOH segment, eight subjects had only a distal LOH segment, two subjects had both proximal and middle LOH segments, 11 subjects had middle and distal LOH segments, two subjects had both proximal and distal LOH segments, five subjects had proximal and distal LOH segments with one having a long 49 Mb LOH segment, three subjects had two LOH segments each and one subject had three separate LOH segments.

One of these PWS subjects (ID #19-221457) with segmental isodisomy 15 also showed an extra X chromosome gain for the entire X chromosome consistent and an XXY male pattern or Klinefelter syndrome along with segmental isodisomy X; therefore, both chromosomes 15 and X were of maternal origin (see Figure 2). Klinefelter syndrome and PWS have also been reported previously as well as trisomy X and PWS indicative of a second female meiotic error during gametogenesis involving both chromosome 15s and the sex chromosome in these individuals [16].

Two PWS subjects (ID #20-068072 and ID #19-207448) had LOHs of 6.21 Mb and 5.57 Mb in size, respectively on chromosome 15. These patterns could support segmental isodisomy in the patients with clinical diagnosis of PWS where the homozygous regions can be smaller in size than 8 Mb without evidence of large ROHs elsewhere in the genome as seen in these two subjects and therefore no evidence of consanguinity which can account for increased number and size of areas of homozygosity [17]. Total isodisomy 15 was seen in four PWS individuals (ID #19-090094, #19-141589, #19-154156, #20-052607) representing an LOH of the entire long arm of chromosome 15 due to errors in female meiosis II.

Maternal heterodisomy 15 or non-deletion status was observed in 24 separate individuals with PWS whereby no cross-over events occurred in female meiosis I or due to a less likely or rare non-deletion (epimutation) involving the imprinting center (IC). Among these 24 heterodisomy/IC defect subjects, four (ID #18-093592, #19-143810, #19-148178, #19-193931) had LOHs of 3 Mb, 3.1 Mb, 4.9 Mb and 3.1 Mb in size, respectively. The other 20 cases showed no LOHs ≥8 Mb or chromosome 15q11-q13 deletions including the imprinting center. DNA microsatellite polymorphic probes from human chromosome 15 would be needed for those not having 15q11-q13 deletions or isodisomy 15 but potentially those with heterodisomy 15 which could resemble non-deletion imprinting center defects via chromosome microarray analysis alone. Therefore, to determine that both 15s are from the mother or maternal disomy 15, biparental (normal) inheritance would indicate an imprinting center defect when examining DNA samples from both parents and the PWS child.

Atypical PWS genetic findings were seen in five individuals with PWS. These included PWS subjects ID #18-085701 involving a pathogenic copy number change on chromosome 15. This small, atypical deletion of chromosome 15 occurred between proximal 15q11.2 breakpoint BP2 and distal 15q breakpoint BP3. The interpretation of this sample showed a female sex pattern by microarray analysis with a small, atypical deletion of approximately 2.3 Mb in size on the long arm of one chromosome 15 at cytogenetic band q11.2-q12 (chr15:23615768-25927232). This heterozygous deletion included the entire SNRPN gene and upstream imprinting centers. The deletion did not include the proximal TUBGCP5, CYFIP1, NIPA2 and NIPA1 genes or distal GABRB3, GABRA5, GABRG3 and OCA2 genes on chromosome 15. This type of deletion is associated with Prader-Willi syndrome when of paternal origin.

**Figure 4:**
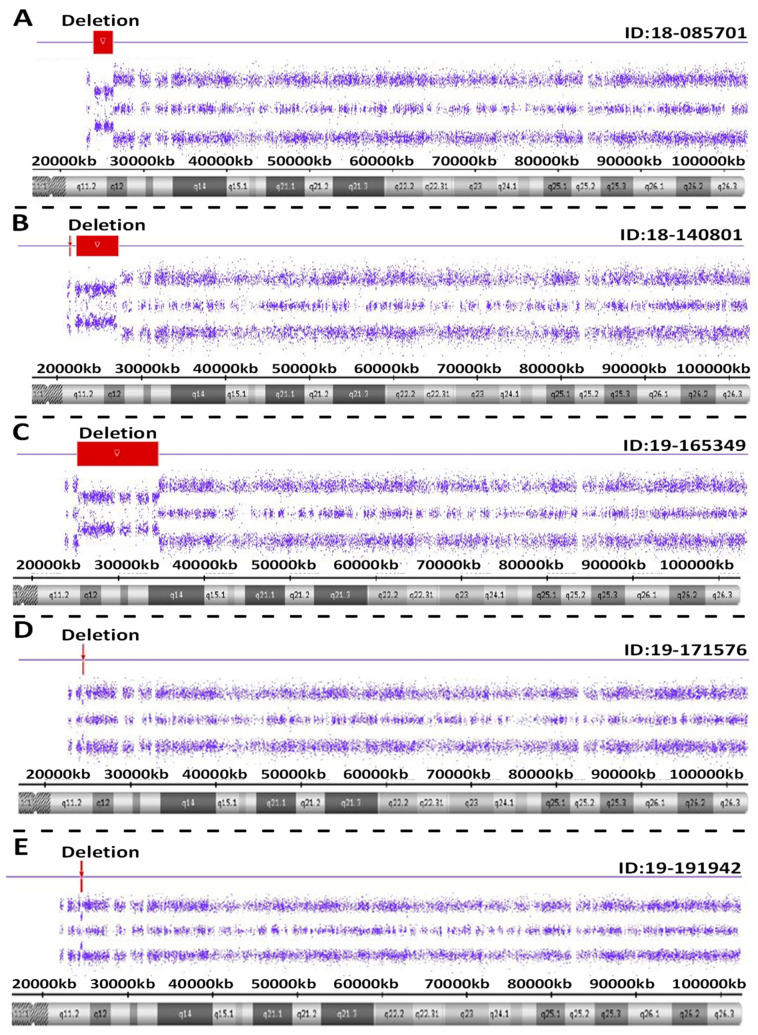
Atypical PWS deletion microarray results with red colored-bars representing the size and location of the deletion on chromosome 15 are shown in five separate PWS participants (**A**–**E**).

For PWS subject ID #18-140801, a pathogenic copy number change was detected on chromosome 15. This atypical deletion found in chromosome 15 included two genes (TUBGCP5 and CYFIP1) located in the 15q11.2 BP1-BP2 region but the two other genes (i.e., NIPA2 and NIPA1) in this region were not deleted. A second deletion was also found at genomic coordinates 23,290,786-28,560,269 involving the proximal long arm of chromosome 15 as typically seen in the 15q11-q13 deletion. Hence, this patient had an atypical deletion pattern not previously reported. Therefore, two different deletions were found, one approximately 195 kb in size involving TUBGCP5 and CYFIP1 genes at genomic coordinates 22,770,421-22,965,401 and a second deletion at 5.2 Mb in size at 15q11.2-q13.1 with coordinates 23,290,786-28,560,269. Due to the rarity of this chromosome deletion pattern, repeat microarray hybridization was undertaken, and the same result was found (see Figure 4). 

For PWS subject ID #19-165349, a pathogenic copy number change was detected on chromosome 15. This atypical deletion in chromosome 15 included the 15q11-q14 region and involved a breakpoint distal to BP2 and included the very distal breakpoint BP5. Hence, the interpretation for this sample showed a male sex pattern by microarray analysis and a large, atypical deletion of approximately 9.42 Mb in size or about 50 percent larger than anticipated for a typical 15q11-q13 deletion. The deletion occurred at cytogenetic bands q11.2-q14 at genomic coordinates chr15:24263392-33680968. This deletion is distal to both the 15q11.2 breakpoint BP2 and the NDN gene, but proximal to the NPAP1 gene. This large deletion included the CHRNA7 gene. A separate atypical large interstitial deletion involving the 15q11-q14 region has been reported previously in a patient by one of the coauthors (i.e., MGB) having an expanded PWS phenotype with findings not typically seen in PWS such as congenital heart defects [18]. 

For PWS subject ID #19-171576, a loss of only one gene PWRN2 (611217) was detected in chromosome 15q11-q13 region. The MS-MLPA or methylation specific testing [19] was required to further confirm genetically the diagnosis of PWS not detectable with microarray analysis. The interpretation of this sample showed a female sex pattern, but no typical 15q11-q13 deletion or maternal segmental or total isodisomy 15. A deletion of approximately 154 kb in size on the long arm of chromosome 15 at cytogenetic band q11.2-q12(chr15:24350855-24504770) was detected. This region included only one gene (PWRN2-Prader-Willi Region Noncoding RNA2) which is poorly characterized and located between the NDN gene and C15orf2 as reported by Buiting et al. [20]. 

For PWS subject ID #19-191942, a pathogenic copy number change on chromosome 15 was seen with a small, atypical deletion including chromosome 15 between proximal 15q11.2 breakpoint BP2 and did not include the distal 15q breakpoint BP3. The interpretation of this sample showed a male sex pattern by microarray analysis and a deletion of approximately 187 kb on the long arm of a chromosome 15 at cytogenetic band q11.2-q12 (chr15:25178112-25365360). This heterozygous deletion included five genes or transcripts including SNRPN, SNHG14, PWAR5, SNORD116-1 and IPW located in the imprinting center region. The deletion did not include the proximal TUBGCP5, CYFIP1, NIPA2 and NIPA1 genes or distal GABRB3, GABRA5, GABRG3 and OCA2 genes on chromosome 15. This type of deletion is apparently associated with Prader-Willi syndrome and the imprinting center.

## 3. Discussion

A high-resolution chromosome microarray analysis was performed on 154 individuals (86 females, 68 males) with Prader-Willi syndrome and PWS molecular genetic classification was determined. Of these individuals 87 showed the typical 15q11-q13 deletion subtypes, 62 showed maternal disomy 15 subclasses and five individuals had an unusual high-resolution chromosome microarray result. One individual was identified having XXY or Klinefelter syndrome in addition to maternal disomy 15. Five individuals with PWS showed atypical chromosome 15 genetic defects including loss of single genes within or outside of the PWSCR, a microdeletion of the imprinting center or surrounding region. Our study is the first to use systematically advanced genetic testing in individuals with PWS clinically diagnosed and entered consecutively in a PWS-specific clinical trial internationally, the largest of its kind, useful for characterizing genetic lesions in a large PWS cohort and their frequencies. This study identified novel atypical genetic lesions of chromosome 15 in those with PWS. These findings will be useful in identifying underlying pathogenesis and disturbed biological pathways in PWS and stimulate further studies on gene-gene-protein interactions needed for development of therapeutic agents, disease surveillance and genetic counseling. 

Maternal disomy 15 is thought to arise from an error in female gametogenesis with the egg containing two chromosome 15 s from the mother, and if fertilized by a normal sperm, then trisomy 15 results in the zygote. Trisomy 15 is lethal and is a relatively common cause of miscarriages in humans. However, if a trisomy 15 rescue event does occur with loss of a chromosome 15 then this leads to a normal 46 chromosome count and the embryo may survive. If the father’s chromosome 15 is lost, then the two remaining chromosome 15s are from the mother leading to maternal disomy 15; hence, a PWS fetus is born. Segmental isodisomy 15 could also be impacted by the presence of centromeric interference preventing recombination near the centromere or mitotic recombination post-zygotically during early cell division in the embryo developing an abnormal clone. This could lead to mosaicism in the developing fetus, potentially impacting clinical involvement. In addition, a 15q11-q13 maternal deletion and uniparental disomy 15 of paternal origin leads to a second genomic imprinting disorder (Angelman syndrome). 

Cytogenetic karyotyping to rule out Robertsonian translocations involving chromosome 15 may also be warranted to assess the chromosome origin and/or presence of marker chromosome 15 s and other rearranged chromosomes. These observations could impact on recurrence risk for subsequent pregnancies and is a limitation of a chromosome microarray analysis only. For example, if the unaffected mother has a 15/15 Robertsonian translocation, trisomy related rescue in the embryo could lead to maternal disomy 15 and PWS or if the unaffected father has this type of translocation and monosomy related rescue of the mother’s chromosome 15 then could lead to PWS. These events may involve segregation in meiosis I and nullisomic events for chromosome 15 in the egg or sperm production. 

The clinical differences occur in those patients with PWS having deletion subtypes when compared with maternal disomy 15 subclasses, particularly those with segmental or total isodisomy 15. They are at a greater risk for unusual or specific clinical findings potentially due to a second genetic disorder, if the unaffected mother is a carrier of a recessive or low penetrant dominant gene allele in the region. A large LOH having more genes in their isodisomic region would increase the likelihood of atypical features and having a second genetic condition. 

There are approximately 600 protein-coding genes recognized on chromosome 15 with 454 annotated in OMIM (www.omim.org) (accessed on 30 August 2022) including 75 autosomal recessive, 44 autosomal dominant and 125 genes for causing clinical disorders. With about 80 Mb of DNA on chromosome 15 and with an average LOH size of 18 Mb in our study in those with maternal segmental isodisomy 15, one would anticipate about 20% of the 600 genes or about 120 would be located in the segmental region and at risk for a second genetic condition along with PWS. Fortunately, humans carry only a very small number of recessive alleles that are considered abnormal. 

Those PWS patients with maternal isodisomy 15 may undergo surveillance depending on the disease-causing genes found in their isodisomic region or when a high index of suspicion arises due to an unexpected phenotype occurs and an altered evolution of the clinical course expected for a patient with PWS. Over 100 autosomal recessive genes have been recognized and localized on chromosome 15 (see Table 1). When dividing the recessive genes on chromosome 15 by chromosome 15 regions (i.e., proximal long arm of chromosome 15- chromosome bands 15q11.2 to 15q14; middle long arm of chromosome 15- chromosome bands 15q15.1 to 15q23 and distal long arm of chromosome 15- chromosome bands 15q24.1 to 15q26.3), there are 14 genes in the proximal long arm region, 57 genes in the middle long arm region with 17 involved with syndrome causation, and 42 genes in the distal long arm region with about 50 percent involved with syndrome causation. For example, Muthusamy et al. [21] summarized the literature regarding maternal disomy 15 and reported a second case of congenital ichthyosis in Prader-Willi syndrome with involvement of the ceramide synthase (*CERS3*) gene on chromosome 15 and two homozygous pathogenic variants in an adult female with PWS having maternal disomy 15. They also summarized the literature and found six reports in PWS patients with maternal disomy 15 with four other disorders with genes on chromosome 15 [Bloom syndrome (BLM gene at 15q26.1), Tay-Sachs disease (HEXA gene at 15q23), deafness-infertility (STRC gene at 15q15.3 and CATSPER2 gene at 15q15.3) and congenital ichthyosis (CERS3 gene at 15q26.3)]. Additionally, data from the ChromosOmics-Database http://cs.tl.de/DB/CA/UPD/0-Start.html [accessed on 14 December 2022] were summarized by L.B. Liehr from Jena, Germany regarding uniparental disomy (UPD) consisting of human case reports from nearly all chromosomes in the medical literature of over 1700 publications. There were greater than 1500 maternal disomy 15 cases with PWS reported with or without clear clinical correlation information in the literature but with normal karyotypes. Eleven of these PWS cases involved a second chromosome 15 gene disorder such as Bloom syndrome, congenital heart defects, congenital ichthyosis and CMT (POLG gene involvement) but no clinical features were characterized for six cases. There were six patients with PWS reported with maternal disomy 15 showing mosaicism ranging between 40% to 90%. There were 26 PWS cases with maternal disomy 15 with or without clear clinical correlation and having balanced karyotypes with 45 chromosomes: three showed der(13;15)(q10;q10)mat; four showed der(14;15)(q10;q10)mat; and 19 showed der(15;15)(q10;q10)mat. Twenty-four cases with PWS had an extra small marker chromosome 15, while 25 PWS cases with maternal disomy 15 involved other chromosome imbalances. These included four patients with PWS and maternal disomy 15 with a 47,XXX karyotype or trisomy X syndrome. Two patients with PWS with maternal disomy 15 also had a 47,XXY karyotype or Klinefelter syndrome. Three patients with PWS with maternal disomy 15 also had a 47,XYY karyotype. Eleven patients with PWS also had an extra chromosome 15 most often identified prenatally. The remaining PWS cases with maternal disomy 15 had other rare chromosomal anomalies or clinical phenotypes. 

A PWS child with segmental isodisomy 15, and particularly those with total isodisomy 15, may therefore need surveillance for disorders or phenotypes associated with disease-causing autosomal recessive genes on chromosome 15 within the isodisomic region as noted. Although the chance that the mother is a carrier of a recessive gene on chromosome 15 is unlikely, there is a chance and may be dependent on the family history. For example, about 2 percent of the general population are carriers of the POLG gene located at 15q26.1 band and leads to mitochondrial DNA depletion syndrome and related disorders [6]. Certain rare genetic disorders are more common in specific populations, e.g., Tay-Sachs disease with gene located at 15q23 and Ashkenazi-Jewish ancestry.

Another genetic phenomenon that can occur in females with PWS and maternal disomy 15 may involve the X chromosome. Females have two X chromosomes, while males have only one X chromosome, but the number of active X-linked genes remain constant in both sexes. This is due to gene dosage compensation as one of the X chromosomes becomes inactivated in females normally in early pregnancy and therefore only one set of X-linked genes are active. The X chromosome inactivation occurs at random and allows for an equal number of active X-linked genes in both sexes. The process of X chromosome inactivation occurs very early in pregnancy and occasionally this process is not random and skewness results. Butler et al. [22] characterized this phenomenon in females with PWS and maternal disomy 15 and showed an overabundance of extreme non-random X chromosome inactivation. Trisomy 15 rescue in early pregnancy of the developing PWS female may allow for a small number of cells to survive and populate embryo development. The small number cells rescued by the trisomic event may have the same X chromosome active leading to non-random X chromosome inactivation skewness in subsequent cell division and presence of an X-linked recessive condition such as colorblindness or hemophilia, if the mother is a carrier of this X-linked disorder but unaffected as a female. Therefore, this X-chromosome skewness can allow for expression of X-linked conditions in PWS females with maternal disomy 15 requiring disease surveillance.

## 4. Materials and Methods

The CytoScan HD array (hg19/GRCh37) consists of 750,000 genotype-able single nucleotides polymorphism (SNP) and 1.9 million non-polymorphic probes performed by Ambry Genetics (Aliso Viejo, CA, USA), a commercial CLIA/CAP genetics testing laboratory on people with PWS who were being screened for enrollment in the DESTINY PWS study. Chromosomal microarray testing was undertaken on buccal swab or peripheral blood samples. The electronic data files were sent to the University of Kansas Medical Center for computer generated data analysis and PWS molecular genetic classification and determination. The cutoff values to detect a deletion included 50 DNA probes occupying at least 20,000 base pairs and a duplication of 30,000 base pairs. Regions of homozygosity (ROH) was set at 3 Mb and loss of heterozygosity (LOH) for determination of the disomic status using ChAS version 3.3.0.139 (r10838) computer software to set at 8 Mb following established protocols or standards [5]. Consanguinity can present a diagnostic problem for detection of segmental uniparental disomy but consanguinity is associated with ROHs in multiple chromosomes and not usually found in abundance in terminal chromosome regions as seen in uniparental disomy [5,6,23]. The total sample in our study consisted of 154 individuals (86 females 68 males), mostly Caucasian with and average age of 13.95 years (range of 4 to 44 years). An image of the microarray data was generated for each participant and a report summarizing the microarray findings with the PWS molecular genetic class was produced.

## 5. Conclusions

In a clinical summary, individuals with PWS and segmental isodisomy 15 or total isodisomy 15 may require further evaluation for additional genetic disorders (e.g., recessive inheritance) where the disease genes would be present in the mother as a carrier status and both identical recessive gene alleles would be passed to the PWS child. Therefore, these PWS children would be at risk for hundreds of conditions where recessive genes are located on chromosome 15 and in the isodisomic region as summarized in Table 1. These children may need close surveillance for these genetic conditions (e.g., Tay-Sachs, Bloom, albinism, hearing loss, seizures, mitochondrial DNA depletion, and others) depending on the genes playing a role in disease causation located in the altered segmental or total isodisomic chromosome 15 regions with input on recurrence risk. In addition, PWS females with maternal disomy 15 may be at risk for X-linked disorders as trisomy 15 rescue occurs in early pregnancy of the disomic 15 female and the X chromosome may therefore be skewed in that PWS female allowing for the presence of X-linked conditions [6]. Healthcare providers may use this review with discussion and be alerted to potential genetic conditions which the PWS child may be at-risk based on their family history and genetic findings as described. More advanced genetic testing may be warranted such as next-generation sequencing of genes on chromosome 15 in both males and females with PWS having maternal segmental or total isodisomy 15 and females should also be screened for X chromosome disorders as noted. 

## Figures and Tables

**Table 1:** Autosomal Recessive Genes and Location on Chromosome 15.

Cytogenetic Location	Genomic Coordinates	Gene Symbol	Phenotype
15q12-q13.1	15:27719008-28099315	*OCA2*	Albinism, brown oculocutaneous, Albinism, oculocutaneous, type II
15q13.1	15:28111040-28322179	*HERC2*	Intellectual developmental disorder
15q13.1	15:29264989-29269822	*NSMCE3*	Lung disease, immunodeficiency, chromosome breakage syndrome
15q13.1-q15.1	15:27800001-42500000	*CILD4*	Ciliary dyskinesia, primary, 4
15q13.3	15:30903852-30943108	*FAN1*	Interstitial nephritis, karyomegalic
15q14	15:34341719-34343136	*NOP10*	Dyskeratosis congenita
15q14	15:36579626-36810244	*CDIN1*	Dyserythropoietic anemia, congenital, type Ib
15q14	15:38488103-38564814	*RASGRP1*	Immunodeficiency 64
15q14	15:34229784-34338057	*SLC12A6*	Agenesis of the corpus callosum with peripheral neuropathy
15q14	15:33400001-39800000	*EIG7*	Epilepsy, juvenile myoclonic
15q15.1	15:40161069-40221123	*BUB1B*	Mosaic variegated aneuploidy syndrome 1
15q15.1	15:42359501-42412317	*CAPN3*	Muscular dystrophy, limb-girdle
15q15.1	15:40520993-40565042	*CCDC32*	Cardiofacioneurodevelopmental syndrome
15q15.1	15:41231268-41281887	*CHP1*	?Spastic ataxia 9, autosomal recessive
15q15.1	15:40470984-40473158	*CHST14*	Ehlers-Danlos syndrome, musculocontractural type 1
15q15.1	15:39934115-40035591	*EIF2AK4*	Pulmonary venoocclusive disease 2
15q15.1	15:40405795-40435947	*IVD*	Isovaleric acidemia
15q15.1	15:40594249-40664342	*KNL1*	Microcephaly 4, primary, autosomal recessive
15q15.1	15:41774484-41827855	*MAPKBP1*	Nephronophthisis 20
15q15.1	15:41387353-41403026	*NDUFAF1*	Mitochondrial complex I deficiency, nuclear type 11
15q15.1	15:40807089-40815084	*ZFYVE19*	Cholestasis, progressive familial intrahepatic, 9
15q15.2	15:42723544-42737128	*CDAN1*	Dyserythropoietic anemia, congenital, type Ia
15q15.2	15:43232590-43266928	*TGM5*	Peeling skin syndrome 2
15q15.2	15:42942897-43106038	*UBR1*	Johanson-Blizzard syndrome
15q15.3	15:43599563-43618800	*STRC*	Deafness, autosomal recessive 16
15q15.3	15:43371101-43409771	*TUBGCP4*	Microcephaly and chorioretinopathy
15q21.1	15:44711517-44718145	*B2M*	Immunodeficiency 43
15q21.1	15:45587123-45609716	*BLOC1S6*	?Hermansky-Pudlak syndrome 9
15q21.1	15:48729083-48811069	*CEP152*	Microcephaly 9, primary, autosomal recessive, Seckel syndrome 5
15q21.1	15:45092650-45114172	*DUOX2*	Thyroid dyshormonogenesis 6
15q21.1	15:45114326-45118421	*DUOXA2*	Thyroid dyshormonogenesis 5
15q21.1	15:45361124-45402227	*GATM*	Cerebral creatine deficiency syndrome 3
15q21.1	15:44665732-44711390	*PATL2*	Oocyte maturation defect 4
15q21.1	15:48206302-48304078	*SLC12A1*	Bartter syndrome, type 1
15q21.1	15:48120990-48142672	*SLC24A5*	Albinism, oculocutaneous, type VI, [Skin/hair/eye pigmentation 4, fair/dark skin]
15q21.1	15:45023195-45077185	*SORD*	Sorbitol dehydrogenase deficiency with peripheral neuropathy
15q21.1	15:45402336-45421415	*SPATA5L1*	Deafness, autosomal recessive 119, Neurodevelopmental disorder with hearing loss and spasticity
15q21.1	15:44562696-44663662	*SPG11*	Amyotrophic lateral sclerosis 5, juvenile, Charcot-Marie-Tooth disease, axonal, type 2X, Spastic paraplegia 11, autosomal recessive
15q21.1	15:45631148-45691281	*SQOR*	Sulfide:quinone oxidoreductase deficiency
15q21.1	15:44956687-44979229	*TERB2*	?Spermatogenic failure 59
15q21.2	15:50907492-51005895	*AP4E1*	Spastic paraplegia 51, autosomal recessive
15q21.2	15:51447791-51622771	*DMXL2*	?Polyendocrine-polyneuropathy syndrome, Developmental and epileptic encephalopathy 81
15q21.2	15:51341655-51413365	*GLDN*	Lethal congenital contracture syndrome 11
15q21.2	15:52115100-52191392	*GNB5*	Intellectual developmental disorder with cardiac arrhythmia, Language delay and ADHD/cognitive impairment with or without cardiac arrhythmia
15q21.2	15:52307283-52529050	*MYO5A*	Griscelli syndrome, type 1
15q21.2	15:50702266-50765706	*SPPL2A*	Immunodeficiency 86, mycobacteriosis
15q21.3	15:55417755-55508234	*DNAAF4*	Ciliary dyskinesia, primary, 25
15q21.3	15:58410554-58569844	*LIPC*	Hepatic lipase deficiency
15q21.3	15:56428724-56465137	*MNS1*	Heterotaxy, visceral, 9, autosomal, with male infertility
15q21.3	15:55319222-55355648	*PIGB*	Developmental and epileptic encephalopathy 80
15q21.3	15:55202966-55289813	*RAB27A*	Griscelli syndrome, type 2
15q21.3	15:53513741-53762878	*WDR72*	Amelogenesis imperfecta, type IIA3
15q22.2	15:63321378-63381846	*CA12*	Hyperchlorhidrosis, isolated
15q22.2	15:59132434-59372871	*MYO1E*	Glomerulosclerosis, focal segmental, 6
15q22.2	15:61852389-62060447	*VPS13C*	Parkinson disease 23, autosomal recessive, early onset
15q22.31	15:63608618-63833948	*HERC1*	Macrocephaly, dysmorphic facies, and psychomotor retardation
15q22.31	15:65001512-65029639	*MTFMT*	Combined oxidative phosphorylation deficiency 15, Mitochondrial complex I deficiency, nuclear type 27
15q22.31	15:64155817-64163022	*PPIB*	Osteogenesis imperfecta, type IX
15q22.31	15:65611350-65661002	*SLC24A1*	Night blindness, congenital stationary (complete), 1D, autosomal recessive
15q22.31	15:65045387-65053397	*SLC51B*	?Bile acid malabsorption, primary, 2
15q22.31	15:64963022-64989914	*SPG21*	MAST syndrome
15q22.31	15:64387836-64455303	*TRIP4*	?Muscular dystrophy, congenital, Davignon-Chauveau type, Spinal muscular atrophy with congenital bone fractures 1
15q23	15:68206992-68257215	*CLN6*	Ceroid lipofuscinosis, neuronal, 6A, Ceroid lipofuscinosis, neuronal, 6B (Kufs type)
15q23	15:72340924-72376014	*HEXA*	GM2-gangliosidosis, several forms, Tay-Sachs disease, [Hex A pseudo deficiency]
15q23	15:71822291-72118600	*MYO9A*	Myasthenic syndrome, congenital, 24, presynaptic
15q23	15:71810554-71818253	*NR2E3*	Enhanced S-cone syndrome
15q24.1	15:72686207-72738473	*BBS4*	Bardet-Biedl syndrome 4
15q24.1	15:74630558-74696024	*EDC3*	?Intellectual developmental disorder, autosomal recessive 50
15q24.1	15:73443164-73560013	*REC114*	Oocyte maturation defect 10
15q24.1	15:74409289-74433958	*SEMA7A*	?Cholestasis, progressive familial intrahepatic, 11
15q24.1	15:74179466-74212259	*STRA6*	Microphthalmia, isolated, with coloboma 8, Microphthalmia, syndromic 9
15q24.1-q24.2	15:74890042-74902219	*MPI*	Congenital disorder of glycosylation, type Ib
15q24.2	15:74919791-74938073	*COX5A*	?Mitochondrial complex IV deficiency, nuclear type 20
15q24.2	15:75355792-75368607	*MAN2C1*	Congenital disorder of deglycosylation 2
15q24.2-q24.3	15:76215353-76311469	*ETFA*	Glutaric acidemia IIA
15q24.3	15:77613027-77820900	*LINGO1*	Intellectual developmental disorder, autosomal recessive 64
15q24.3	15:76347904-76905340	*SCAPER*	Intellectual developmental disorder and retinitis pigmentosa
15q24.3-q25.1	15:77994985-78077711	*TBC1D2B*	Neurodevelopmental disorder with seizures and gingival overgrowth
15q24-q25	15:72400001-88500000	*CILD8*	Ciliary dyskinesia, primary, 8
15q25.1	15:80404382-80597933	*ARNT2*	?Webb-Dattani syndrome
15q25.1	15:78593052-78620996	*CHRNA3*	Bladder dysfunction, autonomic, with impaired pupillary reflex and secondary CAKUT
15q25.1	15:78104606-78131535	*CIB2*	Deafness, autosomal recessive 48, Usher syndrome, type IJ
15q25.1	15:80152789-80186949	*FAH*	Tyrosinemia, type I
15q25.1	15:78149362-78171945	*IDH3A*	Retinitis pigmentosa 90
15q25.1	15:78437431-78501453	*IREB2*	Neurodegeneration, early-onset, with choreoathetoid movements and microcytic anemia
15q25.1	15:80946289-80989819	*MESD*	Osteogenesis imperfecta, type XX
15q25.1	15:79843547-79897285	*MTHFS*	Neurodevelopmental disorder with microcephaly, epilepsy, and hypomyelination
15q25.2	15:82659281-82709875	*AP3B2*	Developmental and epileptic encephalopathy 48
15q25.2	15:82130233-82262734	*EFL1*	Shwachman-Diamond syndrome 2
15q25.2	15:84639285-84654283	*WDR73*	Galloway-Mowat syndrome 1
15q25.3	15:84817356-84873479	*ALPK3*	Cardiomyopathy, familial hypertrophic 27
15q25.3	15:84884662-84975649	*SLC28A1*	[Uridine-cytidineuria]
15q26.1	15:88803436-88875353	*ACAN*	Spondyloepimetaphyseal dysplasia, aggrecan type
15q26.1	15:90717346-90816166	*BLM*	Bloom syndrome
15q26.1	15:90229975-90265759	*CIB1*	Epidermodysplasia verruciformis 3
15q26.1	15:89243979-89317259	*FANCI*	Fanconi anemia, complementation group I
15q26.1	15:89617309-89663049	*KIF7*	?Al-Gazali-Bakalinova syndrome, ?Hydrolethalus syndrome 2, Acrocallosal syndrome, Joubert syndrome 12
15q26.1	15:89776332-89778754	*MESP2*	Spondylocostal dysostosis 2, autosomal recessive
15q26.1	15:89316320-89334824	*POLG*	Mitochondrial DNA depletion syndrome 4A (Alpers type), Mitochondrial DNA depletion syndrome 4B (MNGIE type), Mitochondrial recessive ataxia syndrome (includes SANDO and SCAE), Progressive external ophthalmoplegia, autosomal recessive 1
15q26.1	15:89209869-89221579	*RLBP1*	Bothnia retinal dystrophy
15q26.1	15:90930180-90954093	*UNC45A*	Osteo-oto-hepato-enteric syndrome
15q26.1	15:90998416-91022621	*VPS33B*	Arthrogryposis, renal dysfunction, and cholestasis 1, Cholestasis, progressive familial intrahepatic, 12, Keratoderma-ichthyosis-deafness syndrome, autosomal recessive
15q26.3	15:99971437-100341975	*ADAMTS17*	Weill-Marchesani 4 syndrome, recessive
15q26.3	15:100879831-100916626	*ALDH1A3*	Microphthalmia, isolated 8
15q26.3	15:100400395-100544683	*CERS3*	Ichthyosis, congenital, autosomal recessive 9
15q26.3	15:101175727-101252048	*CHSY1*	Temtamy preaxial brachydactyly syndrome
15q26.3	15:100566924-100602184	*LINS1*	Intellectual developmental disorder, autosomal recessive 27
15q26.3	15:100919357-101078257	*LRRK1*	Osteosclerotic metaphyseal dysplasia

Reference source ‘Online Inheritance In Man’ (www.omim.org)—reviewed online August 30, 2022.

## Data Availability

Reasonable requests available from authors.
